# Cardio-Facio-Cutaneous Syndrome with Precocious Puberty, Growth Hormone Deficiency and Hyperprolactinemia

**DOI:** 10.4274/Jcrpe.1151

**Published:** 2014-03-05

**Authors:** Nurullah Çelik, Peyami Cinaz, Aysun Bideci, Özge Yüce, Hamdi Cihan Emeksiz, Esra Döğer, Orhun Çamurdan

**Affiliations:** 1 Gazi University Medical Faculty, Department of Pediatric Endocrinology, Ankara, Turkey

**Keywords:** Cardio-facio-cutaneous syndrome, MAPK, Precocious puberty, Growth hormone deficiency, hyperprolactinemia

## Abstract

Cardio-facio-cutaneous (CFC) syndrome is a rare disorder characterized by craniofacial dysmorphia, ectodermal abnormalities, cardiac malformations, as well as growth and developmental delay. Although some endocrine abnormalities have been reported in this syndrome, very little is known about CFC syndrome-related endocrine disorders. A 7.5-year-old boy was admitted to our endocrinology clinic with the complaint of short stature. He had a height of 103 cm [-4 standard deviation (SD)], a weight of 16 kg (<3^th^ percentile, -1.7 SD), a facial appearance typical for the CFC syndrome, optic nerve hypoplasia and pulmonary stenosis. Genetic investigation revealed a heterozygous mutation in exon 3 of the *MEK1* gene, c.389A>G (p. Y130C). During his long-term follow-up, the patient developed a variety of endocrine disorders including precocious puberty, growth hormone deficiency and hyperprolactinemia.

## INTRODUCTION

Cardio-facio-cutaneous (CFC) syndrome is a rare, autosomal dominant disorder characterized by multiple congenital anomalies including congenital heart defects, characteristic facial appearance, ectodermal abnormalities, gastrointestinal dysmotility, moderate-to-severe intellectual disability and short stature (1,2). It was first described in 1986 by Reynolds et al (3). The exact prevalence of this syndrome is not known; however, based on the data of CFC International, a family-support group operating worldwide, the estimated number of cases with CFC syndrome is 200-300.

CFC syndrome phenotypically overlaps with Noonan syndrome (NS) and Costello syndrome (CS) since genes that are mutated in all three of these syndromes encode proteins that function in the Ras/mitogen-activated protein kinase (MAPK) signalling pathway and therefore they are all called RASopathies (4). There are four genes associated with CFC; *KRAS*, *BRAF*, *MEK1* and *MEK2* (5,6); approximately 75% of patients with a molecular diagnosis have BRAF mutations (4).

Herein, we present a CFC case due to a previously identified heterozygous mutation in exon 3 of the *MEK1* gene, c.389A>G/p. Y130C (Genetiks Laboratories) (7,8,9) who developed certain endocrinopathies including precocious puberty, hyperprolactinemia and growth hormone (GH) deficiency during his long-term follow-up.

## CASE REPORT

**Figure 1:**
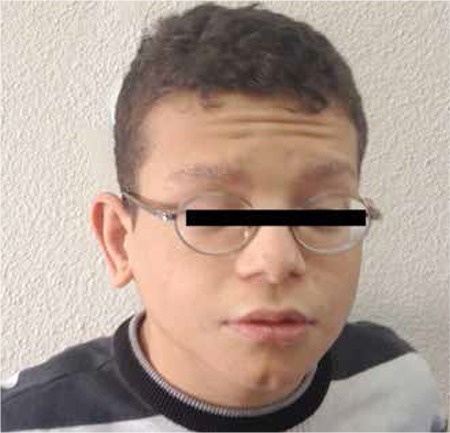
Coarse facial appearance, curly and dry hair, sparse eyebrows, broad nasal tip with anteverted nares

**Figure 2:**
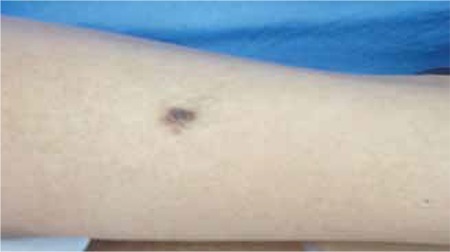
Hairy nevus on the left thigh

**Figure 3:**
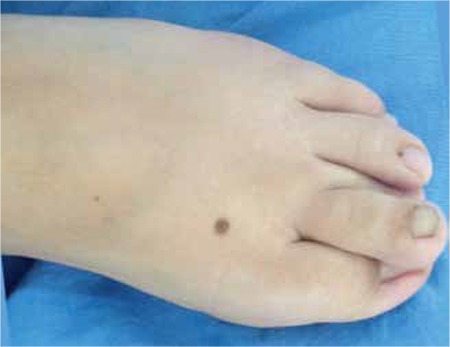
Congenital overlapping toes

A 7.5-year-old boy was admitted to our endocrinology clinic with the complaint of short stature. He was born at 40^th^ gestational week as the first child of a non-consanguineous marriage following a pregnancy complicated by polyhydramnios. His birth weight was 3500 g. On physical examination, his height was 103 cm [-4 standard deviation (SD)] and weight was 16 kg (<3^th^ percentile, -1.7 SD). He had peculiar craniofacial features including curly and dry hair, sparse eyebrows, coarse facial appearance, broad nasal tip with anteverted nares, downslanting palpebral fissures, dysplastic ears, hyperextensible fingers and abnormal skin manifestations such as dry itchy elastic skin, hairy nevus on the left thigh and congenital overlapping toes (Figures 1,2 and 3). His eye examination revealed that he had bilateral optic nerve hypoplasia, intermittent strabismus, ptosis and mild telecanthus. Pulmonary stenosis was detected by echocardiography. His characteristic facial appearance, cardiac anomalies and ophthalmologic findings were in favor of CFC syndrome. Eventually, a heterozygous mutation in exon 3 of the *MEK1* gene, c.389A>G (p. Y130C) detected through his genetic testing confirmed our clinical diagnosis.

In addition to the above-mentioned clinical findings, assessment of pubertal development revealed that there was no axillary or pubic hair development, but remarkably, he had testicular volumes of 6/8 mL (right/left) which were above prepubertal sizes. His basal serum testosterone level was 1.12 ng/mL, luteinizing hormone (LH) level 0.94 IU/L and follicle-stimulating hormone (FSH) level was 4.94 IU/L. After administration of LH-releasing hormone as a provocation test, serum LH and FSH levels rose to peak levels of 17.1 IU/L and 24.69 IU/L, respectively. The clinical and laboratory findings confirmed a diagnosis of central precocious puberty and shortly after, leuprolide acetat treatment was commenced. Magnetic resonance imaging of the brain and pituitary gland was unremarkable. One year after diagnosis of CFC syndrome, it was realized that the patient’s growth velocity was slow with a rate of 3.8 cm/yr and GH stimulation tests using L-dopa and clonidine were performed. Peak GH responses were 2.77 μg/L and 5.06 μg/L, respectively that were suggestive of GH deficiency. After commencing GH replacement therapy at a dose of 25 µg/kg/day, he gained 6 cm at the end of the first year of treatment.

At eleven years of age, the patient was admitted to our endocrinology clinic with a distinct complaint of swelling in his left breast. Laboratory tests performed on two separate days showed that he had elevated prolactin levels of 156.8 and 143.2 ng/mL. His macroprolactin level as well as thyroid function tests were within normal ranges. Repeated MRI of the pituitary gland for prolactinoma was also unremarkable.

## DISCUSSION

Although some endocrine abnormalities have been reported in CFC syndrome, still very little is known about CFC syndrome-related endocrine disorders. During the long-term follow-up of our case, he developed several endocrine disorders including precocious puberty, GH deficiency and hyperprolactinemia.

Delayed puberty is expected in cases with CFC, as is true for other RASopathies, NS and CS, due to their genotype-associated phenotypic similarities (10,11). However, cases with early puberty have also been described in all three of these syndromes (12,13,14). Our patient was a CFC case who developed idiopathic central precocious puberty at age 7.5 years. Contrary to findings typical for early puberty, the bone age of our patient was not advanced and was even delayed (6.5 years at chronological age 7.5 years), as expected in CFC syndrome (15). Armour et al (12) documented a cohort of 38 children with mutation proven CFC. Among these, only one case with precocious puberty was reported, but the distinguishing features of that case were not fully described in that paper.

Short stature is one of the cardinal features of CFC syndrome. Allanson et al (1) reported that two-thirds of patients with CFC had a stature under the 3^rd^ percentile. The underlying cause of short stature in CFC and NS may be similar since both of these syndromes are caused by a germline mutation of the same MAPK pathway that regulates cell differentiation, proliferation and apoptosis (16). Due to the genotypic heterogeneity, GH-secretory dynamics are inconsistent in NS. GH deficiency (45%-37%), neurosecretory dysfunction and completely normal GH secretion were reported in these cases (7). In 2007, the FDA approved GH for treatment of short stature in children with NS (17), but there are no reports concerning GH treatment indication and responsiveness in cases with other RAS/MAPK pathway disorders like CFC syndrome. A retrospectively conducted questionnaire study revealed that two of 38 cases with CFC had GH deficiency (12). In our patient, growth velocity increased to 6 cm/year at the end of the first year of GH treatment in comparison to the rate of 3.8 cm/year prior to GH treatment. Although the first-year GH response was less than expected for a GH-deficient patient, we hesitated to augment the dose of GH in our case due to relatively increased tendency of such cases to develop a cancer in comparison to healthy controls (18). No GH-related side effects were detected in our case during his six-year follow-up.

To our knowledge, this is the first case of CFC syndrome with hyperprolactinemia. Although the association between CFC syndrome and hyperprolactinemia is not clear, we made some speculations regarding mechanisms that may lead to hyperprolactinemia in CFC syndrome. Since a pituitary adenoma had been detected in 10% of patients with idiopathic hyperprolactinemia during a six-year follow-up period (19), likewise, our patient might also have an as yet radiologically undetected microadenoma. Secondly, it has been shown that activation of prolactin receptors induces activation of the MAPK signalling pathway (20,21). Thereby, defects of the MAPK signalling pathway may cause hyperprolactinemia with a feedback mechanism that is as yet unexplained. Lastly, the hyperprolactinemia in our case may have been an incidental finding unrelated to any causal factor.

In conclusion, CFC syndrome may be associated with certain endocrinological disorders. By presenting this case, we aimed to remind our fellow physicians that a comprehensive endocrinological evaluation at diagnosis and a long-term follow-up afterwards are substantial in cases with CFC syndrome.
